# Prevalence and duration of clinical symptoms of pediatric long COVID: findings from a one-year prospective study

**DOI:** 10.3389/fped.2025.1645228

**Published:** 2025-09-22

**Authors:** Vita Perestiuk, Tetyana Kosovska, Liubov Volianska, Oksana Boyarchuk

**Affiliations:** Department of Children’s Diseases and Pediatric Surgery, I. Horbachevsky Ternopil National Medical University, Ternopil, Ukraine

**Keywords:** long COVID, pediatric post-COVID syndrome, SARS-CoV-2, persistent symptoms, ISARIC, children, prospective cohort

## Abstract

**Background:**

Long COVID in children remains a poorly understood condition with wide variability in clinical presentation, duration, and risk factors. **The aim of this study** was to assess the prevalence, spectrum, and duration of long COVID symptoms in pediatric patients following acute SARS-CoV-2 infection using a standardized follow-up tool.

**Methods:**

We conducted a prospective cohort study involving 127 unvaccinated children aged 1 month to 18 years with long COVID according to the WHO definition and confirmed SARS-CoV-2 infection. Participants were followed up at 1–3, 3–6, 6–9, and 9–12 months post-infection using an adapted ISARIC Global Pediatric COVID-19 Follow-Up Questionnaire.

**Results:**

Persistent symptoms of long COVID were reported in 85.8% of patients at 3 months, decreasing to 56.1% at 9 months and 32.5% at 12 months. The most common long-term symptoms included fatigue (52.0%), reduced physical activity (44.1%), and headache (35.3%). Multivariable logistic regression showed that older age was significantly associated with a higher risk of decreased physical activity (OR = 1.51, *p* = 0.038), lack of energy (OR = 2.00, *p* = 0.003), neurological symptoms (OR = 1.86, *p* = 0.007), headache (OR = 4.51, *p* = 0.000), memory impairment (OR = 5.12, *p* = 0.000), difficulty communicating (OR = 4.28, *p* = 0.000), difficulty concentrating (OR = 2.74, *p* = 0.001), cardiological symptoms (OR = 2.34, *p* = 0.022), sensory symptoms (OR = 2.66, *p* = 0.011), and dizziness (OR = 10.02, *p* = 0.034). Younger age was associated with insomnia (OR = 0.49, *p* = 0.018). Female sex was significantly associated with a greater likelihood of lack of energy (OR = 2.55, *p* = 0.048). Hospitalization status was only significantly associated with muscle pain, with outpatients more frequently affected (OR = 0.28, *p* = 0.029).

Overall, 32.5% of all participants continued to experience symptoms of long COVID more than one year acute infection, with fatigue persisting in 19.8%, reduced physical activity in 13.9%, headache in 12.3%.

**Conclusions:**

Long COVID affects children across all age groups and may persist beyond one year in a significant subset. These findings highlight age- and sex-specific symptom profiles and underscore the need for structured pediatric follow-up.

## Introduction

More than five years have passed since the onset of the pandemic caused by severe acute respiratory syndrome coronavirus 2 (SARS-CoV-2), yet an increasing amount of literature continues to shed light on the true prevalence, diversity of clinical manifestations, and long-term consequences of COVID-19 in both adult and pediatric populations. According to the pediatric definition provided by the expert consensus document of the World Health Organization (WHO), long COVID occurs in individuals with a confirmed or probable history of SARS-CoV-2 infection, presenting with symptoms that develop within the first three months following the acute illness, last for at least two months, and impact daily functioning (1). These symptoms may reappear after recovery, persist from the initial illness, and may fluctuate or relapse over time. Additional diagnoses may be identified during evaluation, but this does not rule out the presence of post-COVID syndrome (1).

Long COVID affects people across the globe without exception, regardless of age, racial or ethnic background, sex, pre-existing health conditions, or the course of the SARS-CoV-2 infection (2). It is a complex, non-uniform, multisystem disease characterized by a heterogeneous set of symptoms that can negatively impact virtually any organ system (2, 3).

Several hypotheses have been proposed regarding the pathogenesis of post-COVID syndrome, including the persistence of SARS-CoV-2 reservoirs in tissues, immune dysregulation, reactivation of latent pathogens such as Epstein–Barr virus (EBV) and human herpesvirus 6 (HHV-6), disruptions in specific microbiota, endothelial dysfunction with microthrombus formation, dysfunctional brainstem and/or vagus nerve signaling, and autoimmunity (4–7).

Long COVID is likely a heterogeneous condition with multiple subtypes that may involve different risk factors (genetic and environmental) as well as diverse underlying biological mechanisms, which may also result in varied responses to treatment (2). Factors associated with a higher likelihood of developing long COVID in the pediatric population include female sex, older age (above 10 years), a history of symptomatic COVID-19 infection, hospitalization during the acute phase of illness, prolonged hospital stay, presence of pneumonia and multisystem inflammatory syndrome (MIS) related to SARS-CoV-2, as well as comorbid conditions such as allergic diseases, kidney disorders, diabetes mellitus, obesity, and bronchial asthma (8–10).

The prevalence of long COVID is highly variable and depends on factors such as the study cohort, age, duration of follow-up, comorbid conditions, and COVID-19 vaccination status. According to the World Health Organization, it affects approximately 10%–20% of all individuals who experience acute SARS-CoV-2 infection (11, 12). The frequency of post-COVID syndrome also varies depending on the SARS-CoV-2 variant: it was reported to be 10.8% for the Delta variant and 4.5% for Omicron (13). However, some researchers have noted that more than half of individuals with COVID-19 reported experiencing long-term effects (14, 15). Among children, the prevalence of long symptoms following SARS-CoV-2 infection is also highly diverse, ranging from 0% in a small cohort study conducted in Turkey to 70% among hospitalized patients at a university hospital in Latvia (16, 17).

To date, approximately 200 clinical manifestations of long COVID have been identified, highlighting the multifaceted nature of the condition and its involvement of virtually all organs and systems (5). Roge et al. (18) reported that the most frequently observed complaints in children with COVID-19 included persistent fatigue, cognitive disturbances such as irritability and mood changes, as well as headaches, rhinorrhea, cough, and anosmia/dysgeusia. In contrast, Ashkenazi-Hoffnung et al. (19) identified that common symptoms of post-COVID syndrome include shortness of breath, sleep disturbances, chest pain, paresthesia, and hair loss. According to Bergia et al. (6), frequent manifestations of long COVID included asthenia, headache, myalgia, anxiety, anosmia/ageusia, apathy and sadness, difficulties with concentration, dizziness, and palpitations or tachycardia.

Given the wide variability in the reported prevalence of post-COVID syndrome and the heterogeneity of its clinical presentations, the aim of our study was to assess the prevalence, spectrum, and duration of long COVID symptoms in pediatric patients following acute SARS-CoV-2 infection using a standardized follow-up tool.

## Materials and methods

### Study design and participants

A prospective cohort study was conducted involving a pediatric population with laboratory-confirmed SARS-CoV-2 infection between September 2022 and May 2024 in Ternopil, Ukraine. The study population included children hospitalized at the Pediatric Infectious Disease Department of the Municipal City Hospital No. 2 and Regional Children's Hospital and outpatients who consulted pediatricians regarding COVID-19 or long COVID symptoms.

The participant recruitment process for the study included several stages aimed at identifying and engaging appropriate individuals to take part in the research. First, we clearly defined who exactly needed to be recruited, formulated the objectives, and outlined the expected outcomes. Next, we developed a detailed profile of the target audience.

The inclusion criteria were:

1. Children aged from 1 month to 18 years;
2. Laboratory-confirmed SARS-CoV-2 infection [via polymerase chain reaction (PCR), rapid tests, or serological methods (detection of IgM), which were used interchangeably];
3. Parental or legal guardian consent for participation in the study.

The exclusion criteria were:

1. COVID-19 not confirmed by any diagnostic method;
2. The family's unwillingness to participate in further follow-up;
3. Lack of contact information or inability to reach the family.

The primary recruitment methods included enrolling participants in infectious disease departments or outpatient medical care facilities. During the initial assessment of each patient, we provided information about the study's purpose, objectives, and duration, as well as details regarding confidentiality and data protection. We also clearly outlined the participant requirements.

Upon agreeing to participate in the study and consenting to the use of diagnostic and treatment results in scientific publications, children over 16 years of age or the parents of younger children signed an informed consent form.

The next step involved conducting personal interviews during visits or administering questionnaires via telephone. We assessed both the motivation and interest of candidates, which allowed for a careful selection of participants who best met the study requirements.

To organize each child's participation, logistical support was provided, including the study location and optimal meeting times. All necessary materials and equipment were supplied. The results were analyzed according to several criteria: compliance of data collection with the study protocol, quality of the obtained information, and evaluation of the recruitment process effectiveness, including any modifications made.

We carefully collected general demographic and epidemiological characteristics, as well as vaccination status, existing chronic diseases, and clinical features during the acute phase of SARS-CoV-2 infection, which included disease symptoms, severity, and duration of hospitalization. The severity of COVID-19 was determined according to the WHO definition (20). Based on this, patients were categorized into five groups: asymptomatic, mild, moderate, severe, and critical condition.

Throughout the study, we adhered to all the recommendations of the Declaration of Helsinki of 1975 (revised in 2000). The study was approved by the Ethics Committee of I. Horbachevsky Ternopil National Medical University (Protocol N 70, dated August 1, 2022).

### Long COVID confirmation and follow-up

To identify post-acute symptoms following SARS-CoV-2 infection, we interviewed respondents at intervals of 1–3, 3–6, 6–9, and 9–12 months after hospital discharge. Communication with participants was conducted either by phone—406 (85.1%) or during in-person outpatient visits—71 (14.9%), and were carried out by the same interviewer who was trained according to ISARIC data collection guidelines to ensure consistency. For individuals who did not respond, we attempted to contact them by phone three times before excluding them from the study.

The mode of data collection (phone vs. in-person) did not demonstrate a consistent change across study periods (Supplementary Table S1). However, it was significantly associated with some participant characteristics. Phone interviews were more frequently used for younger children (<6 years) compared with older children (*p* = 0.0005), and for hospitalized participants compared with non-hospitalized ones (*p* < 0.0001). In contrast, in-person interviews were more common for older and non-hospitalized children. Geographic location was not significantly associated with mode of data collection overall, although at 6–9 months urban participants were more likely to be surveyed by phone (*p* = 0.0160). These findings suggest that the choice of data collection mode was influenced more by participant characteristics than by study time point.

For children aged over 8 years, the symptom question was administered directly to the child whenever feasible, with the caregiver present to assist if needed (e.g., clarifying terms). If the child was unable to respond (e.g., due to illness severity), the caregiver provided the answer on their behalf.

In cases where both child and caregiver provided input, the child's report was prioritized, while the caregiver could clarify specific details, such as fever measurement, medication use. We did not systematically capture discrepancies between child and caregiver responses; however, in practice, these were rare and typically resolved immediately during the interview by confirming with both parties to ensure the most accurate response.

We used an adapted questionnaire developed by the International Severe Acute Respiratory and Emerging Infection Consortium (ISARIC)/IP4C Global Pediatric COVID-19 Follow-Up Form. This tool allows for the assessment of physical and psychosocial health, well-being, and their impact on daily functioning, behavior, and relationships. The survey collects data on demographics, existing comorbidities, details of the acute phase of the illness (symptoms and clinical outcomes), and disease severity [hospitalization, intensive care unit (ICU) admission, and oxygen therapy]. Additionally, information is obtained regarding parents' perceptions of changes in their child's emotional and behavioral status, including the perceived causes of these changes (direct or indirect impact of COVID-19, or both), persistent symptoms at the time of follow-up, overall health status compared to before the SARS-CoV-2 diagnosis, and mortality.

Data collection on Long Covid symptoms was based on responses to the question: *“During the past seven days, have you or your child experienced any of the following symptoms that were NOT present before the COVID-19 illness? (If yes, please check them below and indicate their duration).”*

The presence of “long COVID” was determined according to the WHO criteria, defined as the continuation or development of new symptoms at least 3 months after the initial SARS-CoV-2 infection, lasting for at least 2 months with no alternative explanation (1). Patients who exhibited no symptoms during the follow-up period for at least 8 weeks after the onset of acute COVID-19 symptoms were considered fully recovered.

The adaptation and validation of the original English-language ISARIC questionnaire were carried out in six stages:

1. Independent translation from English into Ukrainian by two translators with certified B2-level proficiency in English (according to the Common European Framework of Reference for Languages—CEFRL) and native Ukrainian speakers. One translator had a medical background, and the other did not. Emphasis was placed on conceptual adaptation rather than literal translation.
2. Analysis of the initial translations and creation of a unified “Version 1”.
3. Independent back-translation of “Version 1” from Ukrainian into English by two bilingual translators fluent in English at a minimum of B2 level, without medical education and unfamiliar with the original ISARIC questionnaire.
4. Comparison of “Version 1” and the back-translations by experts from the Department of Pediatrics and Pediatric Surgery at I. Horbachevsky Ternopil National Medical University, followed by the creation of a preliminary draft of the Ukrainian ISARIC questionnaire and a detailed written report on the issues and inaccuracies identified at each stage.
5. Testing of the preliminary version through individual cognitive interviews with 10 patients with long COVID to ensure understanding of the meaning and purpose of each question.
6. Verification of the qualitative characteristics of the pilot testing group in relation to the target population of the questionnaire. The final version of the ISARIC-UA was approved, taking into account the feedback and observations made at each stage.

### Statistical analysis

Statistical processing of the results was performed using the STATISTICA 12 software. A *p*-value of less than 0.05 was considered statistically significant and is highlighted in bold in the tables. Standard methods were used for descriptive statistics: the mean and standard deviation (SD) for normally distributed data; the median and interquartile range (IQR) for skewed distributions; and categorical variables were expressed as frequencies (percentages).

The Mann–Whitney U test was employed for the comparison of absolute values. Frequency indicators were compared using the the chi-square test and Fisher's exact test. Symptom comparison during the post-COVID period was conducted using Cochran's *Q* test, as the analysis involved three or more dependent samples with binary outcomes and a non-normal distribution. Pairwise comparisons between time periods were performed using McNemar's test. To assess the association between age, sex, and hospitalization with long COVID symptoms, multiple logistic regressions was performed using the Benjamini-Hochberg procedure for all comparisons. Analysis of variance (ANOVA) was used to compare parameters across three independent samples.

## Results

### Characteristics of patients with long COVID

During the study period, a total of 245 children with COVID-19 were admitted to the infectious diseases department of regional and city children's hospitals. Among them, 224 met the inclusion criteria. Of these, 190 participants consented to follow-up, while the remaining 34 declined participation. Among the 190 followed-up children, 73 developed long COVID symptoms and were included in the study; the other 117 children had fully recovered.

**Figure 1:**
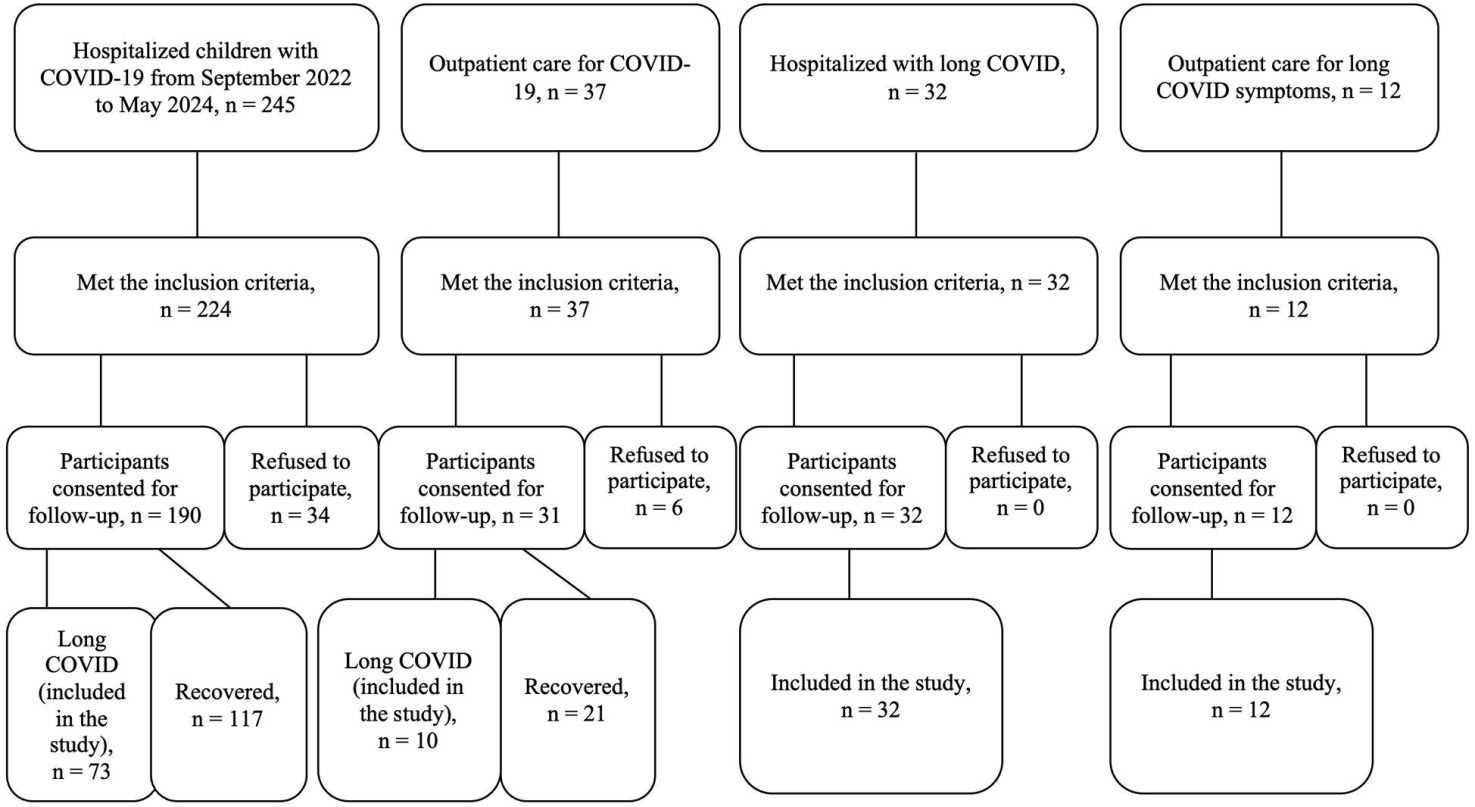
Flowchart of patient enrollment.

The group of non-hospitalized individuals in our study comprised children who had outpatient care for COVID-19 (*n* = 37); were admitted to the hospital due to post-acute sequelae of SARS-CoV-2 infection (*n* = 32) or were consulted at the outpatient department for long COVID symptoms (*n* = 12). Six of them refused to participate, and 54 were included in the study (Figure 1).

A total of 127 children with long COVID and a documented history of acute SARS-CoV-2 infection were examined. Key demographic characteristics, the most common comorbid conditions, and clinical features of the acute phase of coronavirus disease are presented in Table 1 and Supplementary Table S2.

**Table 1:** General characteristics of hospitalized and non-hospitalized patients with long COVID.

Characteristic	Total number, *n* = 127	Hospitalized patients, *n* = 73 (57.5%)		Non-hospitalized patients, *n* = 54 (42.5%)		P1	P2
		Under 6 years, *n* = 50 (68.5%)	Over 6 years, *n* = 23 (31.5%)	Under 6 years, *n* = 17 (31.5)	Over 6 years, *n* = 37 (68.5)		
	Median (interquartile range; IQR) or *n* (%)						
Female gender	70 (55.1)	27 (54.0)	15 (65.2)	12 (70.6)	16 (43.2)	0.2309	0.0977
Age at the time of the initial examination, years	5.2 (1.4; 11.1)	1.1 (0.75; 2.0)	12.9 (8.3; 14.0)	3.0 (1.4; 4.9)	11.1 (7.9; 14.0)	**0.0153**	0.7152
Comorbidities	80 (63.0)	30 (60.0)	17 (73.9)	7 (41.2)	26 (70.3)	0.1776	0.7608
Without comorbidities	47 (37.0)	20 (40.0)	6 (26.1)	10 (58.8)	11 (29.7)	0.1776	0.7608
One comorbidity	49 (38.6)	23 (46.0)	7 (30.4)	5 (29.4)	14 (37.8)	0.2309	0.5589
Two or more comorbidities	31 (24.4)	7 (14.0)	10 (43.5)	2 (11.8)	12 (32.4)	0.8154	0.3880
Number of COVID-19 symptoms	3.0 (2.0; 5.0)	3.0 (3.0; 4.0)	4.0 (3.0; 5.0)	3.0 (2.0; 4.0)	3.0 (2.0; 5.0)	0.1050	**0.0135**
Severity of the acute phase:							
Asymptomatic	8 (6.3)	–	–	3 (17.7)	5 (13.5)	–	–
Mild	101 (79.5)	41 (82.0)	17 (73.9)	13 (76.4)	30 (81.1)	0.6185	0.5123
Moderate	10 (7.9)	4 (8.0)	3 (13.0)	1 (5.9)	2 (5.4)	1.0000	0.3619
Severe	6 (4.7)	4 (8.0)	2 (8.7)	-	-	-	-
Critical	2 (1.6)	1 (2.0)	1 (4.4)	-	-	-	-
Hospitalization in the intensive care unit	8 (6.3)	5 (10.0)	3 (13.0)	-	-	-	-
Oxygen therapy	4 (3.2)	2 (4.0)	2 (8.7)	-	-	-	-
Duration of hospitalization, days	4.0 (3.0; 6.0)	4.5 (3.0; 6.0)	4.0 (3.0; 9.0)	-	-	-	-

P1—comparison between patients in both groups under 6 years old; P2—comparison between patients in both groups over 6 years old.

The Mann–Whitney *U* test was employed for the comparison of absolute values.

For frequency indicators, the chi-square test was applied; however, when the expected frequency was less than 10, Fisher's exact test was used.

Statistically significant values are highlighted in bold.

**Figure 2:**
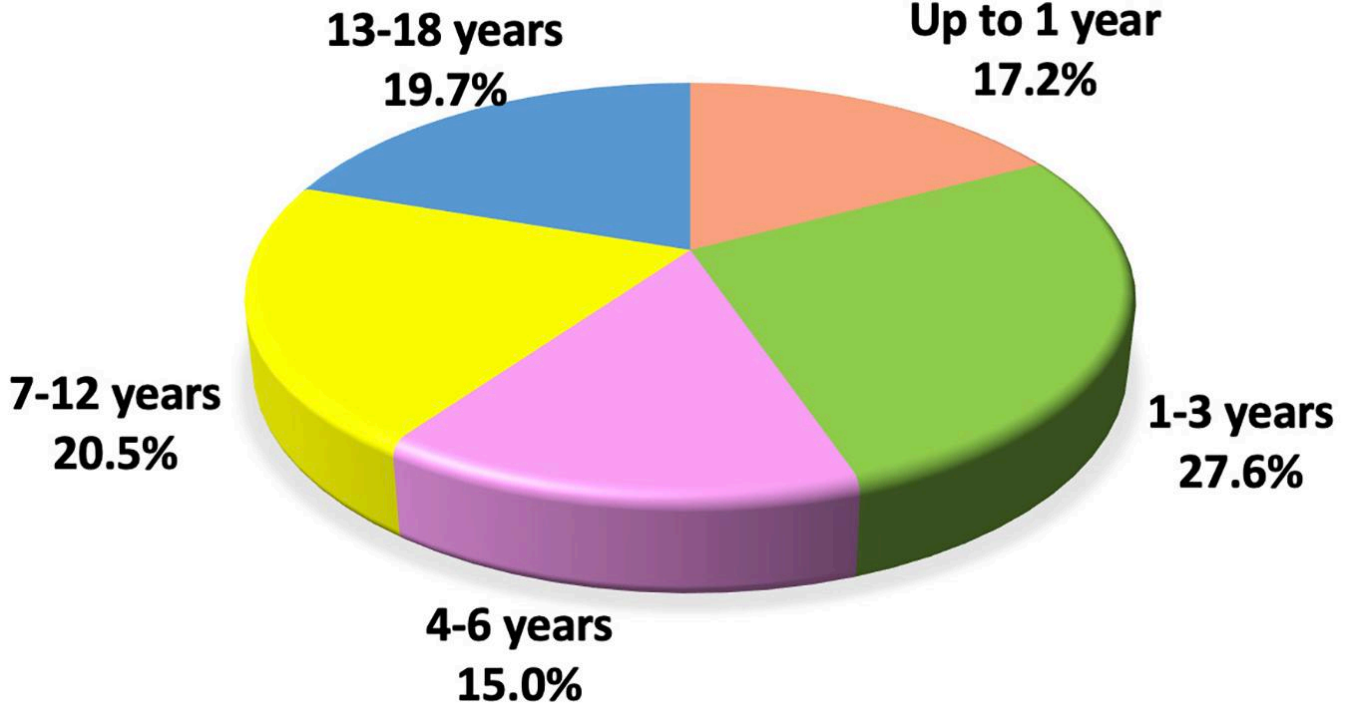
Distribution of patients with long COVID by age.

The median age of the study population was 5.2 years (IQR: 1.4; 11.1), with the highest proportion of cases observed in the 1–3 year age group—27.6% (Figure 2). Girls made up the majority of the cohort at 55.1%. The median number of household members living with each child was 4.0 (IQR: 3.0; 4.0).

Comorbid conditions were identified in 63.0% of the children. Among them, 49 children (38.6%) had one comorbid disease, while 31 (24.4%) had two or more. The most frequent conditions included: allergic rhinitis—18.1% (23/127), overweight or obesity—16.5% (21/127), atopic dermatitis—13.4% (17/127), neurological disorders—10.2% (13/127). Parents of 47 children (37.0%) reported no existing comorbidities. No cases of rheumatologic, hematologic, or genetic disorders, nor of HIV, tuberculosis, anxiety, or depression, were identified in the study cohort.

### Acute SARS-CoV-2 infection symptoms and severity in patients with long COVID

**Figure 3:**
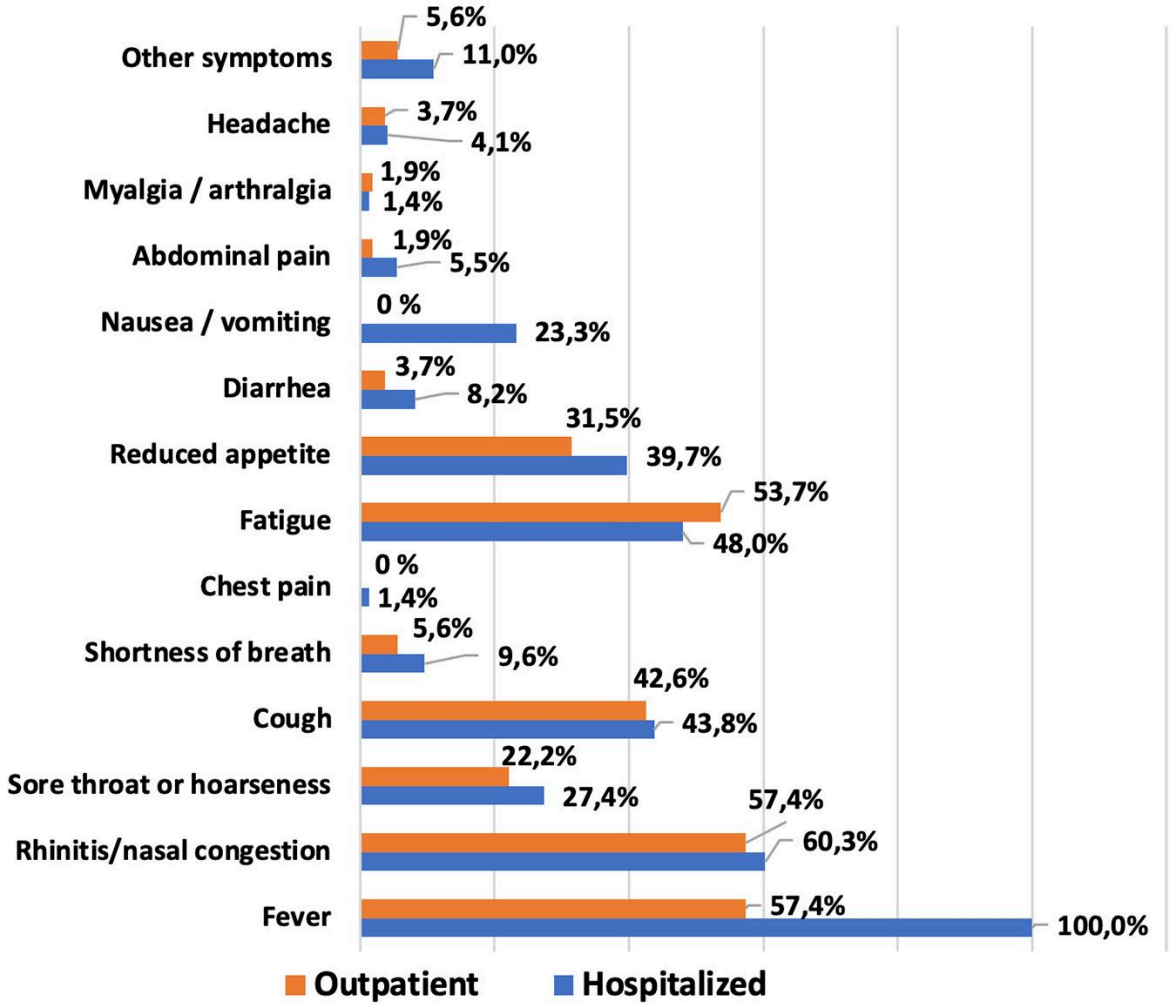
Comparison of COVID-19 symptoms in hospitalized and outpatient children.

The most commonly reported symptoms within the first 14 days of COVID-19 illness in the pediatric population are shown in Figure 3. The most frequent clinical manifestations of acute SARS-CoV-2 infection were: fever—81.9%, rhinitis or congested nose—59.1%, fatigue—50.4%, cough—43.3% and decreased appetite—36.3% (Supplementary Table S2). No cases of anosmia, ageusia, or brain fog were observed among the patients.

In the majority of children, the course of COVID-19 was mild (79.5%). Ten children (7.9%) experienced a moderate course, and eight (6.3%) had severe or critical illness. Eight patients (6.3%) required admission to an intensive care unit (ICU), and four of them (3.2%) received supplemental oxygen. Asymptomatic infection was identified in 8 children (6.3%), diagnosed retrospectively through the detection of SARS-CoV-2 IgG antibodies (Table 1).

### Comparison of COVID-19 clinical characteristics by age between hospitalized and non-hospitalized pediatric patients

Female sex was more common in both groups, with a slight predominance among hospitalized patients (57.5% vs. 51.9%, *p* *=* 0.5244). The median age among children under 6 years of age was higher among outpatients (3.0 vs. 1.1 years, *p* = 0.0153) (Table 1).

No significant difference was found among the two groups of patients in comorbid conditions. A comparison of the most important clinical manifestations between the two cohorts is shown in Figure 3 and Supplementary Table S2. Among the main clinical manifestations of the acute phase of COVID-19, fever was present in 100% of hospitalized children, regardless of age (*p* < 0.0001 and *p* = 0.0001 respectively). Most symptoms were more frequently observed in hospitalized patients, though these differences were not statistically significant. Nausea or vomiting was the only complaint reported exclusively by hospitalized participants, but only in patients older than 6 years the difference was significant (*p* < 0.0001). It was also found that the median number of symptoms was higher in older patients treated in the hospital, and this difference was statistically significant (*p* = 0.0135).

No statistically significant difference was found in the severity of SARS-CoV-2 infection between the two groups.

### Comparison of patient characteristics depending on the method of confirmation of COVID-19

To evaluate whether the method of SARS-CoV-2 confirmation influenced patient characteristics and long COVID outcomes, we compared children diagnosed via PCR, rapid antigen test, or IgM serology (Supplementary Table S3). Significant differences were observed between the groups.

Children with IgM-based confirmation were significantly older than those with PCR or rapid test results (median age: 8.75 vs. 2.25 and 2.35 years, respectively; *p* = 0.0004). Hospitalization was most frequent in PCR-confirmed cases (90.6%) and least common in IgM-confirmed cases (20.0%; *p* < 0.0001).

Fever as an acute COVID-19 symptom was more frequently reported in children with positive PCR or rapid antigen tests (93.8% and 92.0%, respectively) than in the IgM group (64.4%; *p* = 0.0003). This is likely explained by the fact that PCR and rapid test-confirmed children were typically hospitalized in the acute phase of infection when fever was one of the dominant presenting symptoms. In contrast, older children with IgM positivity were often diagnosed at later stages, possibly post-acute, when fever had already subsided.

Age distribution and diagnostic timing also influenced the spectrum of long COVID symptoms. Musculoskeletal, cardiac, respiratory and other symptoms were more commonly reported among older children diagnosed via IgM (26.7%; 15.6%; 17.8%, 11.1%, respectively), compared to those in the PCR and rapid test groups (*p* = 0.0210; *p* = 0.0490, *p* = 0.0011, and *p* = 0.0433, respectively). This may reflect age-related differences in immune response and symptom reporting or underlying pathophysiological mechanisms that differ in post-acute stages.

### Long COVID symptoms and their dynamics based on the ISARIC questionnaire

The average follow-up duration for patients from the time of hospitalization was 10.9 ± 2.0 months. Follow-up interviews were conducted with 127 participants at 1–3 months, 125 participants at 3–6 months, 122 participants at 6–9 months, 101 participants at 9–12 months.

The prevalence of long-term symptoms reported at different follow-up intervals is summarized in Table 2. At 1–3 months, the most commonly reported symptoms were fatigue (52.0%), decreased physical activity (44.1%), and headache (35.3%). By 12 months, these rates declined to 19.8%, 13.9%, and 12.3% respectively (*p* < 0.00001 for all trends).

**Table 2:** Detailed information on long COVID symptoms based on observation time.

Symptoms of long COVID	Post-COVID period				*P*
	1–3 months	3–6 months	6–9 months	9–12 months	
	*n* = 127	*n* = 125	*n* = 122	*n* = 101	
Fatigue	66/127 (52.0)	52/125 (41.6)^∇^^×^	37/122 (30.3)	20/101 (19.8)^ℵ^	**<0** **.** **00001**
General symptoms	76 (59.8)	41 (32.8)^∇^^×^	32 (26.2)	17 (16.8)^ℵ^	**<0** **.** **00001**
Decreased physical activity	56/127 (44.1)*	35/125 (28.0)^∇^^×^	23/122 (18.9)^∞^	14/101 (13.9)^⊕^^ℵ^	**<0** **.** **00001**
Lack of energy	33/125 (26.4)*	25/123 (20.3)^∇^^×^	17/120 (14.2)^∞^	8/100 (8.0)^⊕^^ℵ^	**0** **.** **00003**
Reduced appetite	32/127 (25.2)*	16/125 (12.8)^∇^^×^	9/122 (7.4)^∞^	4/101 (4.0)^⊕^^ℵ^	**<0** **.** **00001**
Fever	2/127 (1.6)*	1/125 (0.8)^∇^^×^	0/122 (0)^∞^	0/101 (0)^⊕^	0.39163
Neurological symptoms	82 (64.6)	61 (48.8)	40 (32.8)	16 (15.8)^ℵ^	**<0** **.** **00001**
Insomnia	27/127 (21.3)*	14/125 (11.2)^∇^^×^	9/122 (7.4)^∞^	1/101 (1.0)^⊕^^ℵ^	**<0** **.** **00001**
Hypersomnia	15/127 (11.8)*	9/125 (7.2)^∇^^×^	6/122 (4.9)^∞^	2/101 (2.0)^⊕^^ℵ^	**0** **.** **00016**
Headache	24/68 (35.3)*	19/68 (27.9)^∇^^×^	12/66 (18.2)^∞^	7/57 (12.3)^⊕^^ℵ^	**<0** **.** **00001**
Memory impairment	16/68 (23.5)*	12/68 (17.7)^∇^^×^	10/66 (15.2)^∞^	5/57 (8.8)^⊕^^ℵ^	**0** **.** **00008**
Emotional excitability	32/127 (25.2)*	26/125 (20.8)^∇^^×^	13/122 (10.7)^∞^	1/101 (1.0)^⊕^^ℵ^	**<0** **.** **00001**
Difficulty communicating	13/64 (20.3)*	9/64 (14.1)^∇×^	6/62 (9.7)^∞^	1/55 (1.8)^⊕^^ℵ^	**0** **.** **00003**
Worsening social relationships	12/83 (14.5)*	7/83 (8.4)^∇^^×^	4/81 (4.9)^∞^	3/69 (4.4)^⊕^^ℵ^	**0** **.** **00021**
Difficulty concentrating	17/75 (22.7)*	11/75 (14.7)^∇^^×^	7/73 (9.6)^∞^	0/64 (0)^⊕^^ℵ^	**<0** **.** **00001**
Musculoskeletal symptoms	19 (15.0)*	16 (12.8)^∇^^×^	12 (9.8)^∞^	6 (6.0)^⊕^^ℵ^	**0** **.** **00214**
Muscle pain	19/68 (27.9)*	16/68 (23.5)^∇^^×^	11/66 (16.7)^∞^	6/57 (10.5)^⊕^^ℵ^	**0** **.** **000007**
Joint pain or swelling	4/127 (3.2)*	3/125 (2.4)^∇×^	2/122 (1.6)^∞^	0/101 (0)^⊕^^ℵ^	0.26147
Gastrointestinal symptoms	15 (11.8)*	7 (5.6)^∇^^×^	4 (3.3)^∞^	2 (2.0)^⊕^^ℵ^	**0** **.** **00010**
Abdominal pain	5/68 (7.4)*	3/68 (4.4)^∇^^×^	1/66 (1.5)^∞^	0/57 (0)^⊕^^ℵ^	**0** **.** **01537**
Nausea	3/68 (4.4)*	2/68 (2.9)^∇^^×^	2/66 (3.0)^∞^	2/57 (3.5)^⊕^^ℵ^	0.39163
Constipation	10/127 (7.9)*	4/125 (3.2)^∇×^	1/122 (0.8)^∞^	0/101 (0)^⊕^^ℵ^	**0** **.** **00006**
Cardiological symptoms	10 (7.9)*	8 (6.4)^∇^^×^	6 (4.9)^∞^	2 (2.0)^⊕^^ℵ^	**0** **.** **00106**
Tachycardia	8/127 (6.3)*	7/125 (5.6)^∇×^	5/122 (4.1)^∞^	2/101 (2.0)^⊕^^ℵ^	**0** **.** **00559**
Bradycardia	2/127 (1.6)*	1/125 (0.8)^∇×^	1/122 (0.8)^∞^	0/101 (0)^⊕^^ℵ^	0.26147
Respiratory symptoms	9 (7.1)*	5 (4.0)^∇×^	3 (2.5)^∞^	2 (2.0)^⊕^^ℵ^	**0** **.** **00182**
Cough	5/127 (3.9)*	5/125 (4.0)^∇×^	3/122 (2.5)	2/101 (2.0)^⊕^^ℵ^	0.06117
Shortness of breath	4/127 (3.2)*	0/125 (0)^∇^^×^	0/122 (0)^∞^	0/101 (0)^⊕^^ℵ^	**0** **.** **00738**
Sensory symptoms	11 (8.7)*	9 (7.2)^∇^^×^	7 (5.7)^∞^	3 (3.0)^⊕^^ℵ^	**0** **.** **00106**
Vision problems	5/127 (3.9)*	3/125 (2.4)^∇×^	3/122 (2.5)^∞^	2/101 (2.0)^⊕^^ℵ^	0.09648
Disturbances in smell and/or taste	2/64 (3.1)*	2/64 (3.1)^∇^^×^	1/62 (1.6)^∞^	1/55 (1.8)^⊕^^ℵ^	0.39163
Paresthesia	4/64 (6.3)*	4/64 (6.3)^∇^^×^	3/62 (4.8)^∞^	0/55 (0)^⊕^^ℵ^	**0** **.** **01923**
Other symptoms	6 (6.6)*	2 (2.2)^∇^^×^	2 (2.3)^∞^	1 (1.3)^⊕^^ℵ^	**0** **.** **00810**
Balance problems	3/91 (3.3)*	0/91 (0)^∇^^×^	0/88 (0)^∞^	0/76 (0)^⊕^^ℵ^	**0** **.** **02929**
Dizziness	4/91 (4.4)*	2/91 (2.2)^∇^^×^	2/88 (2.3)^∞^	1/76 (1.3)^⊕^^ℵ^	0.09648

* *p* ≤ 0.05 when comparing periods 1–3 months and 3–6 months.

^∞^ *p* ≤ 0.05 when comparing periods 1–3 months and 6–9 months.

^⊕^ *p* ≤ 0.05 when comparing periods 1–3 months and 9–12 months.

^∇^ *p* ≤ 0.05 when comparing periods 3–6 months and 6–9 months.

^×^ *p* ≤ 0.05 when comparing periods 3–6 months and 9–12 months.

^ℵ^ *p* ≤ 0.05 when comparing periods 6–9 months and 9–12 months.

*P* values were calculated using the Cochran's test, as there were 3 or more dependent samples with binary values and non-normal distribution.

Values *p* ≤ 0.05 were considered statistically significant and are highlighted in bold.

Comparisons between periods were performed using the McNemar test.

Symptom clustering by system showed that 64.6% of participants experienced neurological symptoms, 59.8% general symptoms, 15.0% musculoskeletal, 11.8% gastrointestinal, 7.9% cardiac, 7.1% respiratory, 8.7% sensory, and 6.6% balance-related symptoms. Over 62.2% of children reported symptoms from more than one system.

Neurological symptoms such as insomnia (21.3% → 1.0%), memory impairment (23.5% → 8.8%), emotional excitability (25.2% → 1.0%), and concentration difficulties (22.7% → 0%) declined significantly during the study period (*p* < 0.0001). Musculoskeletal complaints (primarily muscle pain) also decreased from 27.9% to 10.5%, *p* < 0.00001.

No cases of nasal congestion/rhinorrhea, fainting, altered consciousness, diarrhea, vomiting, or skin rash were reported at any timepoint. Some symptoms, such as bradycardia, joint pain, and shortness of breath, were rare and resolved within the first 6 months.

We found that at 6 months post-COVID-19, 14.0% of children had fully recovered, at 9 months, this number increased to 43.9%, by 12 months, 67.5% of patients no longer reported long COVID symptoms. However, by the end of the 12-month follow-up, 32.5% of children continued to experience at least one symptom of long COVID. Fatigue (19.8%), reduced physical activity (13.9%), and headache (12.3%) remained the most persistent, followed by muscle pain (10.5%), memory impairment (8.8%), and lack pf energy (8%).

**Figure 4:**
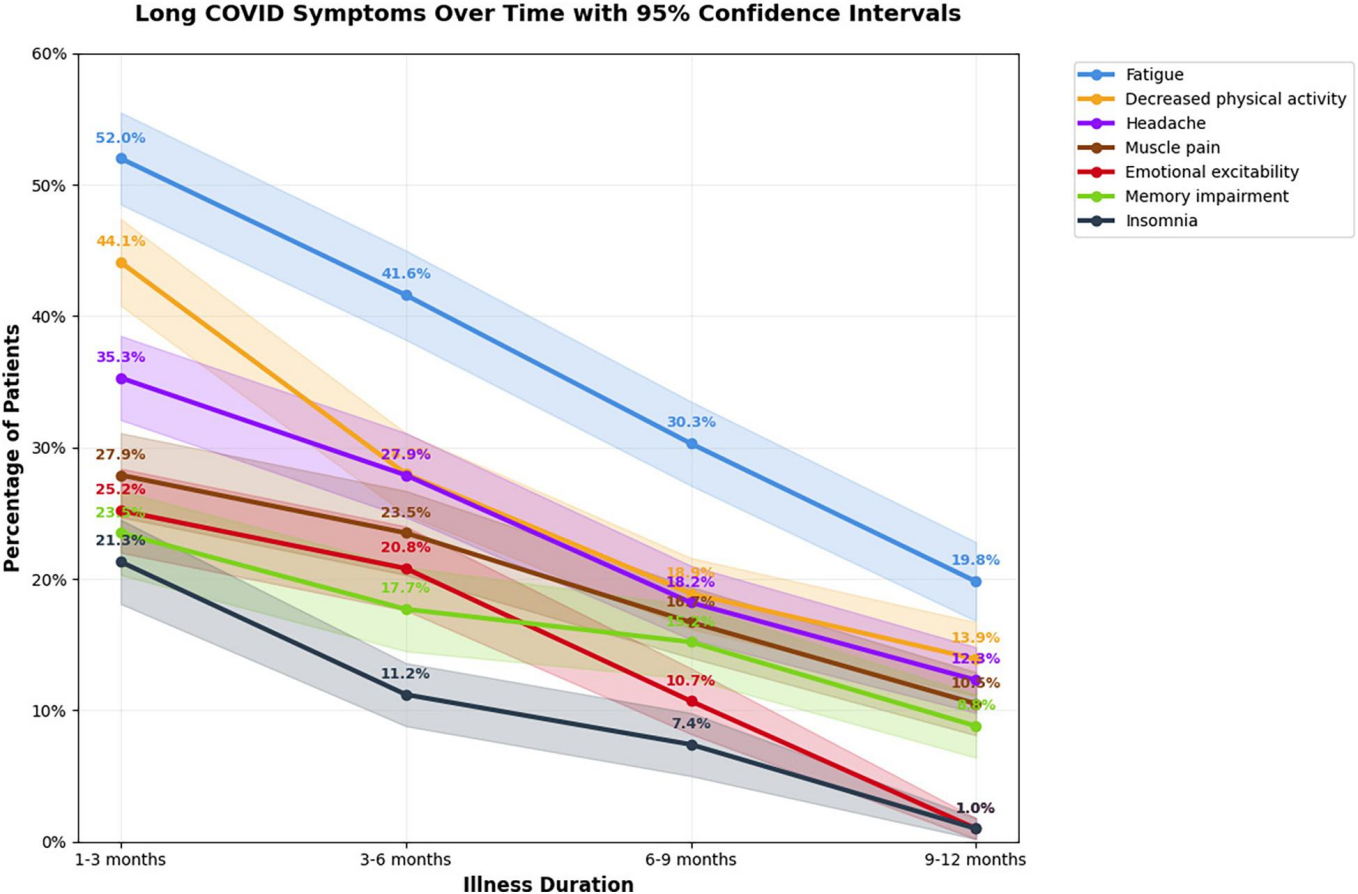
Duration and prevalence of the most common long COVID symptoms in children over time, presented with 95% confidence intervals. The radar plot shows the proportion of children reporting persistent symptoms at 1–3, 3–6, 6–9, and 9–12 months after acute COVID-19 infection. Each colored line represents a different observation period, and shaded areas illustrate 95% confidence intervals.

**Figure 5:**
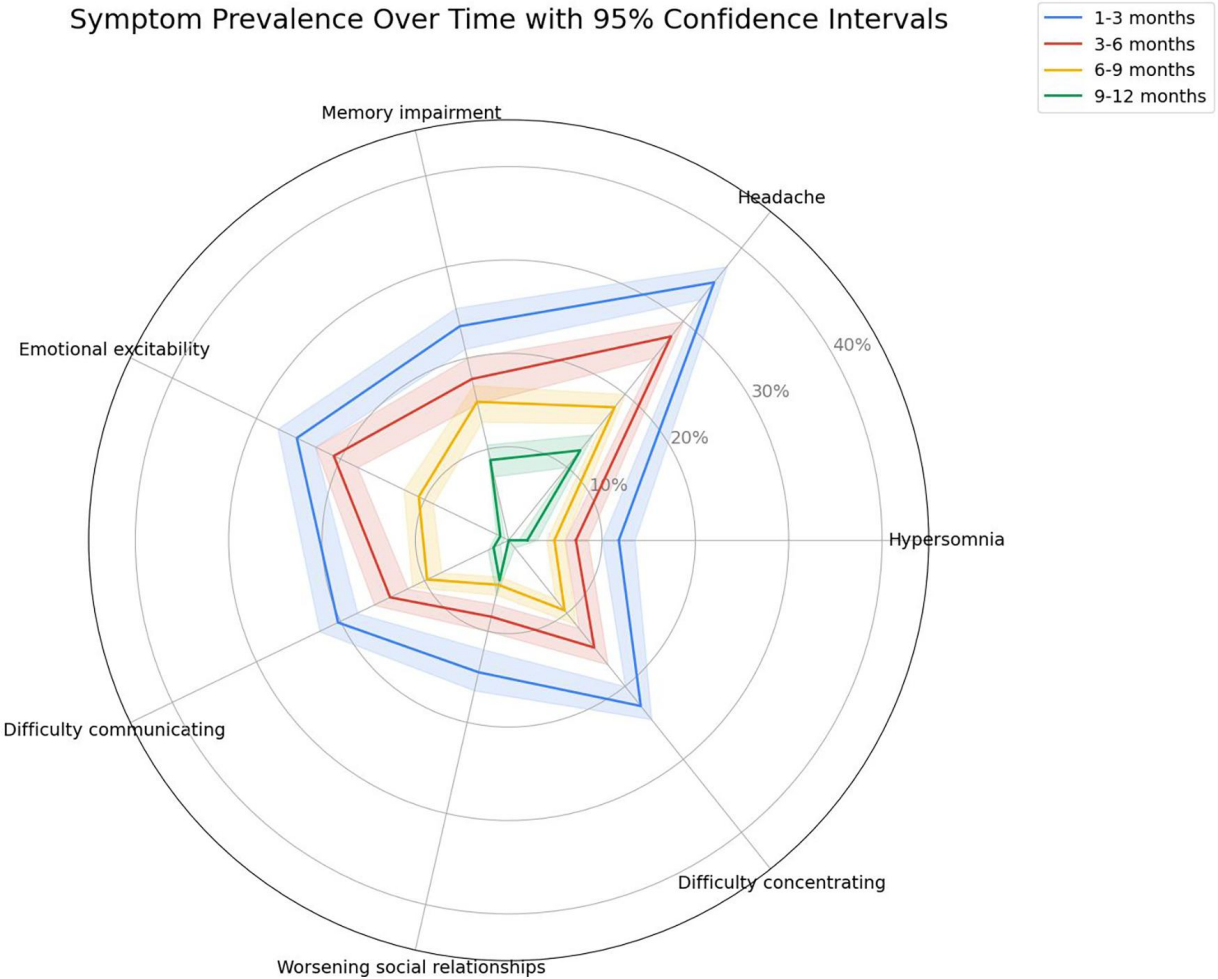
Comparison of the frequency of general and neurological manifestations of long COVID in children over time, with 95% confidence intevals. The line graph illustrates the prevalence of the most common long COVID symptoms at 1–3, 3–6, 6–9, and 9–12 months following acute infection. Each colored line represents one symptom, while shaded areas correspond to 95% confidence intervals.

The dynamics of the most common post-COVID clinical manifestations and comparison of the frequency of general and neurological manifestations of long COVID depending on the time of observation are shown in Figures 4, 5, respectively.

### Long COVID symptoms depend on age

In children under the age of 6, the most common long-term effects were fatigue (49.3%), myalgia (44.4%), decreased appetite (37.3%), reduced physical activity (35.8%), insomnia (32.8%), and emotional lability (23.9%). Among children over the age of 6, the most frequently reported symptoms were fatigue (55.0%), reduced physical activity (53.3%), lack of energy (40.7%), headache (39.0%), memory impairment (27.1%), difficulty concentrating (27.1%), emotional lability (26.7%), and myalgia (25.4%).

Multivariable logistic regression (Table 3) showed that older age was significantly associated with a higher risk of decreased physical activity (OR = 1.51, *p* = 0.038), lack of energy (OR = 2.00, *p* = 0.003), neurological symptoms (OR = 1.86, *p* = 0.007), headache (OR = 4.51, *p* = 0.000), memory impairment (OR = 5.12, *p* = 0.000), difficulty communicating (OR = 4.28, *p* = 0.000), difficulty concentrating (OR = 2.74, *p* = 0.001), cardiological symptoms (OR = 2.34, *p* = 0.022), sensory symptoms (OR = 2.66, *p* = 0.011), and dizziness (OR = 10.02, *p* = 0.034). Younger age was associated with insomnia (OR = 0.49, *p* = 0.018).

**Table 3:** Results of multiple logistic regression assessing associations between age, sex, and hospitalization with symptoms of long COVID.

Symptoms of long COVID	Age (OR, 95% CI, *p*)	Hospitalization (OR, 95% CI, *p*)	Sex (OR, 95% CI, *p*)
Fatigue	1.19 (0.81–1.73), *p* = 0.364	0.68 (0.32–1.46), *p* = 0.326	0.85 (0.42–1.73), *p* = 0.652
General symptoms	1.00 (0.68–1.47), *p* = 1.000	0.61 (0.28–1.34), *p* = 0.219	0.80 (0.39–1.64), *p* = 0.538
Decreased physical activity	**1.51** **(1.02–2.22), *p*** **=** **0.038**	0.56 (0.26–1.22), *p* = 0.146	1.38 (0.66–2.89), *p* = 0.392
Lack of energy	**2.00** **(1.28–3.14), *p*** **=** **0.003**	0.44 (0.18–1.10), *p* = 0.081	**2.55** **(1.01–6.43), *p*** **=** **0.048**
Reduced appetite	0.67 (0.42–1.08), *p* = 0.102	1.47 (0.59–3.68), *p* = 0.413	0.75 (0.33–1.70), *p* = 0.485
Fever	1.49 (0.32–6.98), *p* = 0.616	0.00 (0.00–0.00), *p* = 0.997	0.94 (0.06–16.01), *p* = 0.968
Neurological symptoms	**1.86** **(1.19–2.92), *p*** **=** **0.007**	1.54 (0.67–3.54), *p* = 0.312	1.09 (0.51–2.32), *p* = 0.825
Insomnia	**0.49** **(0.28–0.88), *p*** **=** **0.018**	1.60 (0.57–4.46), *p* = 0.370	1.57 (0.63–3.90), *p* = 0.335
Hypersomnia	1.47 (0.85–2.54), *p* = 0.168	2.85 (0.79–10.27), *p* = 0.109	1.61 (0.51–5.11), *p* = 0.417
Headache	**4.51** **(2.40–8.47), *p*** **=** **0.000**	0.77 (0.26–2.30), *p* = 0.638	0.49 (0.17–1.47), *p* = 0.204
Memory impairment	**5.12** **(2.41–10.90), *p*** **=** **0.000**	1.94 (0.54–6.93), *p* = 0.307	0.42 (0.12–1.47), *p* = 0.176
Emotional excitability	1.08 (0.71–1.66), *p* = 0.708	1.14 (0.48–2.73), *p* = 0.771	1.78 (0.77–4.09), *p* = 0.178
Difficulty communicating	**4.28** **(1.95–9.37), *p*** **=** **0.000**	0.87 (0.23–3.24), *p* = 0.830	0.88 (0.24–3.19), *p* = 0.842
Worsening social relationships	**1.95** **(1.03–3.71), *p*** **=** **0.042**	0.53 (0.14–1.99), *p* = 0.345	0.54 (0.16–1.90), *p* = 0.340
Difficulty concentrating	**2.74** **(1.51–4.96), *p*** **=** **0.001**	0.61 (0.19–1.93), *p* = 0.400	0.89 (0.29–2.72), *p* = 0.842
Musculoskeletal symptoms	1.52 (0.89–2.59), *p* = 0.122	**0.28** **(0.09–0.87), *p*** **=** **0.029**	0.73 (0.26–2.03), *p* = 0.543
Muscle pain	1.52 (0.89–2.59), *p* = 0.122	**0.28** **(0.09–0.87), *p*** **=** **0.029**	0.73 (0.26–2.03), *p* = 0.543
Joint pain or swelling	1.00 (0.33–3.01), *p* = 0.999	0.00 (0.00–0.00), *p* = 1.000	0.92 (0.12–7.09), *p* = 0.939
Gastrointestinal symptoms	0.96 (0.53–1.73), *p* = 0.896	1.55 (0.46–5.26), *p* = 0.481	0.66 (0.22–1.97), *p* = 0.461
Abdominal pain	1.72 (0.70–4.19), *p* = 0.235	1.71 (0.25–11.95), *p* = 0.586	0.49 (0.08–3.12), *p* = 0.451
Nausea	2.10 (0.61–7.23), *p* = 0.239	0.62 (0.05–7.94), *p* = 0.717	0.40 (0.03–4.64), *p* = 0.461
Constipation	0.31 (0.09–1.03), *p* = 0.056	0.86 (0.18–4.10), *p* = 0.851	0.81 (0.22–3.04), *p* = 0.755
Cardiological symptoms	**2.34** **(1.13–4.82), *p*** **=** **0.022**	0.48 (0.11–2.08), *p* = 0.326	0.81 (0.21–3.15), *p* = 0.762
Tachycardia	2.11 (0.98–4.53), *p* = 0.057	0.68 (0.14–3.23), *p* = 0.628	0.80 (0.18–3.49), *p* = 0.765
Bradycardia	3.32 (0.48–22.75), *p* = 0.223	0.00 (0.00–0.00), *p* = 0.991	0.92 (0.05–16.45), *p* = 0.953
Respiratory symptoms	1.14 (0.54–2.40), *p* = 0.740	0.21 (0.04–1.19), *p* = 0.078	0.40 (0.09–1.72), *p* = 0.219
Cough	1.08 (0.41–2.81), *p* = 0.878	0.52 (0.07–3.73), *p* = 0.518	0.55 (0.09–3.42), *p* = 0.520
Shortness of breath	1.19 (0.38–3.71), *p* = 0.768	0.00 (0.00–0.00), *p* = 0.998	0.29 (0.03–2.95), *p* = 0.294
Sensory symptoms	**2.66** **(1.25–5.66), *p*** **=** **0.011**	0.24 (0.05–1.21), *p* = 0.084	0.67 (0.17–2.56), *p* = 0.555
Vision problems	1.62 (0.63–4.22), *p* = 0.319	0.23 (0.02–2.31), *p* = 0.213	1.36 (0.21–8.75), *p* = 0.747
Disturbances in smell and/or taste	3.08 (0.45–21.31), *p* = 0.253	0.00 (0.00–0.00), *p* = 1.000	0.00 (0.00–0.00), *p* = 1.000
Paresthesia	3.91 (1.00–15.27), *p* = 0.050	0.47 (0.04–5.05), *p* = 0.532	0.80 (0.10–6.46), *p* = 0.836
Other symptoms	**6.13** **(1.55–24.30), *p*** **=** **0.010**	0.71 (0.11–4.66), *p* = 0.719	1.72 (0.26–11.26), *p* = 0.570
Balance problems	3.23 (0.68–15.43), *p* = 0.142	0.00 (0.00–0.00), *p* = 0.999	1.99 (0.16–24.96), *p* = 0.593
Dizziness	**10.02** **(1.19–84.59), *p*** **=** **0.034**	1.75 (0.19–15.92), *p* = 0.620	2.47 (0.22–28.09), *p* = 0.466

OR, odds ratio; 95% CI, 95% confidence interval; Age, in years (continuous variable); Hospitalization, 1 = hospitalized, 0 = outpatient care; Sex, 1 = female, 0 = male.

R-adjusted *p*-values were calculated using the Benjamini–Hochberg procedure across all comparisons.

Associations with FDR-corrected *p* ≤ 0.05 were considered statistically significant and are highlighted in bold.

### Long COVID symptoms depend on gender

Among all clinical manifestations of long COVID in girls, the most prevalent were fatigue (50.0%), reduced physical activity (47.1%), lack of energy (33.8%), emotional lability (30.0%), headache (29.0%), insomnia (24.3%), myalgia (23.7%), decreased appetite (22.9%), and difficulty concentrating (22.5%). Boys more frequently reported fatigue (54.4%), headache (43.3%), reduced physical activity (40.4%), myalgia (33.3%), memory impairment (30.0%), reduced appetite (28.1%), and difficulty concentrating (22.9%).

Multivariable logistic regression (Table 3) showed that female sex was significantly associated with a greater likelihood of lack of energy (OR = 2.55, *p* = 0.048).

### Long COVID symptoms depend on hospitalization during acute SARS-CoV-2 infection

In hospitalized pediatric patients, the most common long-term manifestations of COVID-19 were fatigue (46.6%), reduced physical activity (35.6%), headache (34.6%), memory impairment (30.8%), and decreased appetite (30.1%). Among outpatient children, the most frequently reported symptoms were fatigue (59.3%), reduced physical activity (55.6%), lack of energy (37.0%), headache (35.7%), and myalgia (33.3%).

Multivariable logistic regression (Table 3) revealed that hospitalization status was significantly associated with muscle pain, with outpatients being more frequently affected (OR = 0.28, *p* = 0.029).

## Discussion

In our study, the majority of hospitalized patients were children under 6 years of age, whereas among non-hospitalized patients, those over 6 years of age predominated. Floyd et al. (21) also demonstrated that a significant number of hospitalized patients were younger, while individuals older than 17 years tended to prefer outpatient care (*p* = 0.0071 and *p* = 0.0009, respectively).

The prevalence of comorbid conditions did not differ significantly between the two groups in our study. In contrast, researchers from the United States of America found that comorbidities were less common among outpatients with COVID-19 (*p* < 0.0001), whereas hospitalized patients were more likely to have two or more serious comorbidities (*p* < 0.0001) (21). Fernández-de-Las-Peñas et al. (22) also noted that the frequency of comorbid conditions, especially with more than three comorbidities, was higher among hospitalized patients (61.3% vs. 44.8% and 29.6% vs. 16.1%). Pérez-González et al. (23) indicated that comorbid conditions were more than twice as common among hospitalized individuals.

An analysis of symptoms of long COVID showed that the most common of them were fatigue (52.0%), reduced physical activity (44.1%), headache (35.3%), sleep disturbances (33.1%), muscle pain (27.9%). Other studies, including our previous research, also confirm that among all long-term consequences, the most frequently observed in children was fatigue (24–27). In contrast, a study involving a pediatric population conducted in Israel showed that the most common post-COVID symptom was headache (28).

By carefully analyzing all symptoms according to the age of the children, we found that younger age was associated with insomnia, while older age was significantly associated with a higher risk of decreased physical activity, lack of energy, headache, memory impairment, difficulty communicating, worsening social relationship, difficulty concentrating, and dizziness. Buonsenso et al. (29) reported that children under the age of 10 more frequently experienced persistent cough and constipation, while children over the age of 10 were more commonly affected by fatigue, headache, insomnia, myalgia, difficulty concentrating/confusion, joint pain or swelling, chest pain, nausea, and disturbances in smell and taste. Other researchers have reported that in children aged 6–11 years, the features of post-COVID-19 syndrome were stomach pain, itchy skin or rash, sleep problems, nausea, or vomiting, while in adolescents aged 12–17 years, changes or loss of sense of smell and/or taste, body, muscle, or joint pain, fatigue, drowsiness, and low energy levels (30). The Danish researchers also noted variability in symptoms across children of different ages. They noted that patients aged 0–5 years were more likely to suffer from fatigue, while school-aged children were more likely to report a loss of smell and taste (24).

During the study, we also found that a lack of energy was significantly more common in women. In a research involving the adult population, using the ISARIC questionnaire, it was reported that women more frequently experienced persistent fatigue and shortness of breath, and also had significantly poorer overall health status (31). A study conducted in Milan found that women more frequently experienced dyspnea, fatigue, anosmia, dysgeusia, gastrointestinal symptoms, brain fog, as well as symptoms of depression and anxiety (32). Marcilla-Toribio et al. (33) reported that hair loss was more commonly observed in women compared to men. According to Shah et al. (34), palpitations and hair loss were more commonly observed among female individuals as part of the symptom profile of long COVID. In contrast, scientists studying the symptoms of long COVID among children in Italy did not find any differences among them depending on gender (35).

Comparing clinical manifestations between the two cohorts, we found that muscle pain was significantly more common in non-hospitalized patients. Findings from other researchers, however, have varied. Roge et al. (18) reported that cough, rhinorrhea, speech disturbances, and changes in body weight were more prevalent in hospitalized children, with statistically significant differences. However, Chasapidou et al. (36) showed that non-hospitalized patients exhibited the same long-term symptoms as hospitalized ones even one year post-infection — fatigue, general weakness, dyspnea, and tachycardia. Meanwhile, Spanish researchers observed that myalgia, fatigue, brain fog, and sleep disturbances occurred significantly more often in hospitalized children, whereas anosmia was more frequent in outpatients (23).

The duration of symptoms varied, but fatigue, reduced physical activity, lack of energy, elevated temperature, neurological complaints, muscle pain, tachycardia, and cough lasted the longest, more than 12 months in 32.5% of children. However, we observed a steady decline in the prevalence of long-term effects over time. In contrast, a nationwide cohort study conducted in Denmark found that, depending on age, symptoms disappeared in at least 54%–75% of children within 1–5 months (24). Huang et al. (37) found that in adults, fatigue or muscle weakness, sleep problems, anxiety, or depression were common even six months after symptom onset. Another study using an online survey found similar results. The authors noted that 65.2% of adult patients had symptoms lasting at least 180 days, and the probability of their duration exceeding 35 weeks was 91.8% (95% confidence interval 89.5% to 93.5%), with no statistically significant difference between genders (38). Ranucci et al. (39) analyzed the determinants of major physical (fatigue, fever, cough, shortness of breath, headache, sleep disturbances) and neuropsychological (anxiety, depression, memory dysfunction, brain fog) symptoms and found that starting from a 95% persistence rate three months after hospital discharge, physical manifestations remained present in 82% of patients after one year and in 45% of patients after 18 months, decreasing to approximately 10% only after 20 months. After one year of follow-up, patients had neuropsychological complaints in 59% of cases; after 18 months, they persisted in 28% of individuals. This is confirmed by a recent study by researchers from the United States, which found that 90%-100% of people usually recover within three years, but approximately 5% still experience symptoms that do not completely subside (40).

### Strengths and limitations of the study

The strengths of this study include the fact that we conducted a detailed investigation of long COVID in the pediatric population. A comparison was made between the main clinical characteristics of acute SARS-CoV-2 infection in hospitalized and outpatient children. Only unvaccinated children with a primary infection were included, which makes the results valid. Most participants were surveyed four times using the ISARIC questionnaire at different periods after the initial illness, making the study more thorough and of higher quality. The topic is highly relevant, yet data remains limited, as most studies focus on specific categories of patients and only certain parameters of their health and functioning.

There are some limitations of the study. First, we did not include a control group of children who had never had COVID-19. However, at present, it is likely difficult to find such a cohort, as most of the population has been infected with SARS-CoV-2. All participants were selected from a tertiary-level pediatric hospital, meaning they may differ from patients treated at other pediatric healthcare facilities. Another limitation of our study is that we did not assess potential differences in symptom reporting between child self-reports and parent proxy reports, nor did we conduct a reliability assessment between these reporting modes. This may have impacted the consistency of reporting for subjective symptoms such as fatigue and emotional lability. A further limitation of our study is that although we recorded the reporting source (child, parent, or both), we did not conduct a systematic analysis to evaluate whether differences existed between self- and proxy-reported symptom data, which may have introduced reporting variability, particularly for subjective symptoms. An additional limitation of our study is the use of both in-person and phone follow-up interviews, which may have introduced variability in responses. However, we used the standardized ISARIC Paediatric COVID-19 follow-up questionnaire in both modes to ensure consistency. We acknowledge that some subgroup analyses in our study were based on small cell sizes (<10 participants), which may limit the statistical validity and generalizability of these specific findings. Additionally, as our study used all available data within a defined cohort, we did not perform *a priori* sample size calculation or power analysis, which we recognize as a limitation. Future studies with larger sample sizes are needed to validate these subgroup findings.

## Conclusions

According to the results of the study, the most common symptoms of long COVID are fatigue, decreased physical activity, and headache. Older age is associated with a higher risk of decreased physical activity, lack of energy, headache, memory impairment, difficulty communicating, difficulty concentrating, cardiological symptoms, sensory symptoms, and dizziness. Younger age was associated with insomnia. Female sex is associated with lack of energy.

Overall, 32.5% of all participants continued to experience symptoms of long COVID more than one year after their initial illness.

The results confirm the development of long COVID in pediatric patients of all ages, regardless of the severity and course of the acute illness. Studying the impact of post-COVID-19 syndrome on children's health and daily functioning is an important goal that requires special attention in the field of healthcare.

## Data Availability

The raw data supporting the conclusions of this article will be made available by the authors, without undue reservation.
