# Supplementary material for: Prevalence and duration of clinical symptoms of pediatric long COVID: findings from a one-year prospective study
Source: Front Pediatr. 2025 Sep 22;13:1645228. doi: 10.3389/fped.2025.1645228 (PMC12499359; doi:10.3389/fped.2025.1645228)
Supplement: Supplementary file 1 [file Table1.docx]

**Table S1.** Association between data collection method and participant characteristics

| Characteristic | 1-3 months | | 3-6 months | | 6-9 months | | 9-12 months | | Total | |
| --- | --- | --- | --- | --- | --- | --- | --- | --- | --- | --- |
|  | phone | in-person | phone | in-person | phone | in-person | phone | in-person | phone | in-person |
| Age  Under 6 years    Over 6 years | 56/67 (83.6)  38/60 (63.3) | 11/67 (16.4)  22/60 (36.7) | 56/65 (86.1)  45/60 (75.0) | 9/65 (13.9)  15/60  (25.0) | 61/63 (96.8)  56/59 (94.9) | 2/63 (3.2)  3/59 (5.1) | 49/50 (98.0)  43/51 (84.3) | 1/50 (2.0)  8/51 (15.7) | 222/245  (90.6)  182/230  (79.1) | 23/245  (9.4)  48/230  (20.9) |
| p | **0.0094** | | 0.1137 | | 0.5949 | | **0.0158** | | **0.0005** | |
| Hospitalization status  Hospitalized    Non-hospitalized | 66/73 (90.4)  28/54 (51.9) | 7/73 (9.6)  26/54 (48.1) | 65/73 (89.0)  36/52 (69.2) | 8/73 (11.0)  16/52 (30.8) | 70/71 (98.6)  47/51 (92.2) | 1/71 (1.4)  4/51 (7.8) | 53/56 (94.6)  39/45 (86.7) | 3/56 (5.4)  6/45 (13.3) | 254/273  (93.0)  150/202 (74.3) | 19/273  (7.0)  52/202 (25.7) |
| p | **<0.0001** | | **0.0056** | | 0.0770 | | 0.1620 | | **<0.0001** | |
| Geographic location  Urban area    Rural area | 66/86 (76.7)  28/41 (68.3) | 20/86 (23.3)  13/41 (31.7) | 69/85 (81.2)  32/40 (80.0) | 16/85 (18.8)  8/40 (20.0) | 83/84 (98.8)  34/38 (89.5) | 1/84 (1.2)  4/38 (10.5) | 62/67 (92.5)  30/34 (88.2) | 5/67 (7.5)  4/34 (11.8) | 280/322 (87.0)  124/153 (81.0) | 42/322 (13.0)  29/153 (19.0) |
| p | 0.3099 | | 0.8762 | | **0.0160** | | 0.4733 | | 0.0914 | |

Chi-square was used to calculate p-values.

Statistically significant values are highlighted in bold.
