# Supplementary material for: Prevalence and duration of clinical symptoms of pediatric long COVID: findings from a one-year prospective study
Source: Front Pediatr. 2025 Sep 22;13:1645228. doi: 10.3389/fped.2025.1645228 (PMC12499359; doi:10.3389/fped.2025.1645228)
Supplement: Supplementary file 2 [file Table2.docx]

**Table S2.** Detailed information about general characteristics of hospitalized and non-hospitalized patients with long COVID

| Characteristic | Total number,  n = 127 | Hospitalized patients,  n = 73 | | Non-hospitalized patients,  n = 54 | | | P1 | P2 |
| --- | --- | --- | --- | --- | --- | --- | --- | --- |
|  |  | Under 6 years,  n = 50 | Over 6 years,  n = 23 | Under 6 years,  n = 17 | | Over 6 years, n = 37 |  |  |
|  | Median (interquartile range; IQR) or n (%) | | | | | |  |  |
| Comorbidities  Neurological conditions / neurodisability  Gastrointestinal problems  Heart diseases  Bronchial asthma  Allergic rhinitis  Food allergies  Atopic dermatitis  Immune system disorders  Kidney problems  Overweight or obesity  Malnutrition  Endocrine diseases, excluding diabetes  Without comorbidities  One comorbidity  Two or more comorbidities | 80 (63.0)  13 (10.2)  7 (5.5)  7 (5.5)  5 (3.9)  23 (18.1)  9 (7.1)  17 (13.4)  1 (0.8)  6 (4.7)  21 (16.5)  11 (8.7)  2 (1.6)  47 (37.0)  49 (38.6)  31 (24.4) | 30 (60.0)  5 (10.0)  0  1 (2.0)  0  3 (6.0)  5 (10.0)  10 (20.0)  0  2 (40.0)  7 (14.0)  4 (8.0)  0  20 (40.0)  23 (46.0)  7 (14.0) | 17 (73.9)  3 (13.0)  3 (13.0)  1 (4.4)  2 (8.7)  8 (34.8)  3 (13.0)  3 (13.0)  0  1 (4.4)  4 (17.4)  4 (17.4)  0  6 (26.1)  7 (30.4)  10 (43.5) | 7 (41.2)  1 (5.9)  0  1 (5.9)  1 (5.9)  3 (17.7)  0  2 (11.8)  0  1 (5.9)  1 (5.9)  1 (5.9)  0  10 (58.8)  5 (29.4)  2 (11.8) | 26 (70.3)  4 (10.8)  4 (10.8)  4 (10.8)  2 (5.4)  9 (24.3)  1 (2.7)  2 (5.4)  1 (2.7)  2 (5.4)  9 (24.3)  2 (5.4)  2 (5.4)  11 (29.7)  14 (37.8)  12 (32.4) | | 0.1776  1.0000  -  0.4460  0.2537  0.1660  0.3186  0.4443  -  1.0000  0.6687  1.0000  -  0.1776  0.2309  1.0000 | 0.7608  1.0000  1.0000  0.6401  0.6335  0.3956  0.1525  0.3619  1.0000  1.0000  0.7486  0.1911  0.5192  0.7608  0.5589  0.3880 |
| COVID-19 symptoms:  Fever ≥ 38°C  Rhinitis / nasal congestion  Sore throat or hoarseness  Cough  Shortness of breath  Chest pain  Fatigue  Reduced appetite  Diarrhea  Nausea / vomiting  Abdominal pain  Myalgia / arthralgia  Headache  Other symptoms | 104 (81.9)  75 (59.1)  32 (25.2)  55 (43.3)  10 (7.9)  1 (0.8)  64 (50.4)  46 (36.3)  8 (6.3)  17 (13.4)  5 (3.9)  2 (1.6)  5 (3.9)  12 (9.5) | 50 (100.0)  31 (62.0)  9 (18.0)  24 (48.0)  4 (8.0)  0  20 (40.0)  20 (40.0)  4 (8.0)  8 (16.0)  2 (4.0)  0  1 (2.0)  6 (12.0) | 23 (100.0)  13 (56.5)  11 (47.8)  8 (34.8)  3 (13.0)  1 (4.4)  15 (65.2)  9 (39.1)  2 (8.7)  9 (39.1)  2 (8.7)  1 (4.4)  2 (8.7)  3 (13.0) | 11 (64.7)  12 (70.6)  0  6 (35.3)  1 (5.9)  0  9 (52.9)  7 (41.2)  1 (5.9)  0  0  0  0  0 | 20 (54.1)  19 (51.4)  12 (32.4)  17 (46.0)  2 (5.4)  0  20 (54.1)  10 (27.0)  1 (2.7)  0  1 (2.7)  1 (2.7)  2 (5.4)  3 (8.1) | | **<0.0001**  0.5235  0.0982  0.3628  1.0000  -  0.3522  0.9319  1.0000  0.1034  1.0000  -  1.0000  0.3252 | **0.0001**  0.6963  0.2331  0.3938  0.3619  0.3833  0.3938  0.3271  0.5524  **<0.0001**  0.5524  1.0000  0.6335  0.6662 |

P1 – comparison between patients in both groups under 6 years old; P2 – comparison between patients in both groups over 6 years old;

For frequency indicators, the chi-square test was applied; however, when the expected frequency was less than 10, Fisher’s exact test was used

Statistically significant values are highlighted in bold
