# Supplementary material for: Prevalence and duration of clinical symptoms of pediatric long COVID: findings from a one-year prospective study
Source: Front Pediatr. 2025 Sep 22;13:1645228. doi: 10.3389/fped.2025.1645228 (PMC12499359; doi:10.3389/fped.2025.1645228)
Supplement: Supplementary file 3 [file Table3.docx]

**Table S3.** Comparison of patient characteristics depending on the method of confirmation of COVID-19

| **Characteristic** | **Rapid antigen test** | **PCR** | **IgM** | **P** |
| --- | --- | --- | --- | --- |
|  | n = 50 | n = 32 | n = 45 |  |
| Female, n (%) | 31 (62.0) | 17 (53.1) | 21 (46.7) | 0.3215 |
| Age, years | 2.35 (1.0; 7.0) | 2.25 (0.71; 13.0) | 8.75 (5.25; 13.0) | **0.0004** |
| Geographic location  Urban area  Rural area | 37 (74.0)  13 (26.0) | 18 (56.2)  14 (43.8) | 31 (68.9)  14 (31.1) | 0.2398 |
| Hospitalized | 35 (70.0) | 29 (90.6) | 9 (20.0) | **<0.0001** |
| Symptoms of COVID-19  Fever  Respiratory symptoms  Gastrointestinal symptoms  Severe fatigue  Decreased appetite  Other | 46 (92.0)  40 (80.0)  10 (20.0)  28 (56.0)  22 (44.0)  6 (12.0) | 30 (93.8)  20 (62.5)  8 (25.0)  10 (31.3)  10 (31.3)  3 (9.4) | 29 (64.4)  36 (80.0)  5 (11.1)  26 (57.8)  14 (31.1)  5 (11.0) | **0.0003**  0.1372  0.2683  **0.0428**  0.3395  0.9335 |
| Long COVID symptoms  Fatigue  General  Neurological  Musculoskeletal  Gastroenterological  Cardiological  Respiratory  Sensory  Other | 21 (42.0)  24 (48.0)  26 (52.0)  5 (10.0)  3 (6.0)  1 (2.0)  0 (0)  3 (6.0)  1 (2.0) | 16 (50.0)  18 (56.3)  24 (75.0)  2 (6.3)  7 (21.9)  2 (6.3)  1 (3.1)  3 (9.4)  0 (0) | 29 (64.4)  31 (68.9)  29 (64.4)  12 (26.7)  5 (11.1)  7 (15.6)  8 (17.8)  5 (11.1)  5 (11.1) | 0.0887  0.1191  0.1034  **0.0210**  0.0906  **0.0490**  **0.0011**  0.7233  **0.0433** |

Chi-square or Fisher's exact test (for values less than 10) in 3x2 format was used to calculate p-values because the data were binary and the distribution was normal. Age was compared using analysis of variance (ANOVA).
