# Supplementary material for: Prevalence and duration of clinical symptoms of pediatric long COVID: findings from a one-year prospective study
Source: Front Pediatr. 2025 Sep 22;13:1645228. doi: 10.3389/fped.2025.1645228 (PMC12499359; doi:10.3389/fped.2025.1645228)
Supplement: Supplementary file 4 [file Datasheet1.docx]

**Bioethical Document Approved by**
**The Academic Council of**
**I.Ya. Horbachevsky Ternopil National Medical University**
**of the Ministry of Health of Ukraine**
"___" ________________, 2024

**Informed Voluntary Consent of the Patient**

**for the Use of Diagnostic and Treatment Results in Scientific Research**

I, _____________________________________________________________, have received information from __________________________________________________________________________

(name of the medical institution)

about the nature of my (or my child’s) illness, its course, diagnostics, and treatment.

I am familiar with the examination and treatment plan.
I have received a full explanation of the nature and purpose of the diagnostic and treatment process and I give my consent to the physician _________________________________________
to use the results of diagnostics and treatment in scientific research, without disclosing my personal data.

I, ____________________________________________________________________, confirm all of the above with my signature ______________.

"___" ________________, 2024
