# Supplementary material for: Prevalence and duration of clinical symptoms of pediatric long COVID: findings from a one-year prospective study
Source: Front Pediatr. 2025 Sep 22;13:1645228. doi: 10.3389/fped.2025.1645228 (PMC12499359; doi:10.3389/fped.2025.1645228)
Supplement: Supplementary file 5 [file Datasheet2.docx]

**APPROVED**
Order of the Ministry of Health of Ukraine
September 8, 2022, No. 1621

**Assessment of the quality of life and psychological state of children**

**with long COVID-19 in conditions of martial law**

1. **Abstract**

The course of COVID-19 in children is accompanied by a negative impact on their physical health, but the consequences for their mental health, well-being, and overall quality of life pose an even more serious issue and are likely to persist much longer. Disruptions to educational and recreational routines, combined with concerns about family income and health, are causing many young people to experience fear, anger, and anxiety about their future. These challenges have been significantly worsened under martial law, as children’s already difficult lifestyles changed abruptly. Forced isolation, online learning, lack of communication with peers, and inability to attend various sports activities and favorite clubs have been further compounded by prolonged stays in shelters, forced displacement, loss of loved ones, separation from family members, and uncertainty about the future. The COVID-19 pandemic under martial law may serve as a trigger for serious health conditions in children in the future. Currently, there is no data in Ukraine on the prevalence of long COVID-19 among children, nor have the characteristics of long COVID-19 during wartime been studied.

A prospective cohort study involving 300 children under the age of 18 diagnosed with COVID-19 is planned. An adapted questionnaire from the International Severe Acute Respiratory and Emerging Infection Consortium (ISARIC) titled "Paediatric COVID-19 Health and Wellbeing Follow Up" will be used for follow-up assessments at 1, 3, 6, and 12 months after illness to diagnose symptoms of long COVID-19 and assess its course. Children with symptoms of long COVID-19 will undergo thorough examinations to detect possible complications. The "Pediatric Quality of Life Inventory-4.0-Core-All (PedsQL™ 4.0) questionnaire" will be used to evaluate the impact of long COVID-19 on children’s quality of life.

In addition, risk factors for the development of long COVID-19 will be identified. For this purpose, the course of acute illness, presence of comorbid conditions, vitamin D and zinc status will be analyzed.

Identifying clinical and psychological symptoms of long COVID-19 in children will enable timely diagnosis of the condition, while quality-of-life assessment will reveal the medical and social impacts of SARS-CoV-2 infection on children's lives. Determining risk factors will allow for the implementation of timely preventive and therapeutic-rehabilitation measures to improve children’s quality of life and prevent the development of serious illnesses in the future.

**2. Research issues and the current state of research worldwide**

**1) The problem addressed by the project**

The COVID-19 pandemic has had a detrimental impact on both the physical and mental health of many individuals worldwide, including children. Although in most cases COVID-19 manifests mildly in children, the risk of complications remains. While most children recover completely, some develop a range of long-term effects such as fatigue, concentration difficulties, anxiety, and sleep disturbances. Some children also experience psychological consequences after having COVID-19. These symptoms can persist after the acute illness or appear after the initial recovery. Certain health disorders may last for several months after the acute phase of the disease.

The World Health Organization has introduced the term post-COVID-19 condition (long COVID-19), defined as a condition occurring in individuals with a history of probable or confirmed SARS-CoV-2 infection, usually within three months of the onset of COVID-19, with symptoms and effects lasting for at least two months and not explainable by an alternative diagnosis. Early signs of health disorders in children often go unnoticed by parents, who may not seek medical assistance.

The increasing incidence of COVID-19 adds strain on healthcare systems and, under the chronic stress of war, may lead to more severe and prolonged consequences for children's health.

**2) Review of research findings on the issue and unresolved questions (with references to specific publications)**

According to the European Centre for Disease Prevention and Control, most children either do not develop symptoms of coronavirus infection or experience only mild ones. However, both symptomatic and asymptomatic children can experience long-term consequences months after the initial infection. The most common symptoms include fatigue, insomnia, anxiety, myalgia, arthralgia, shortness of breath, palpitations, dizziness, and cognitive and psychological disorders (Thomson H., 2021). Two months after COVID-19 onset, 80% of patients reported headaches, concentration difficulties, dizziness, and muscle weakness (Ludvigsson, 2020). Overall, about 44% of patients noted a decline in quality of life after recovering from COVID-19 (Yong, 2021). The development of long-term symptoms was not always associated with the severity of the acute COVID-19 infection (Boyer, 2022). Children reported persistent symptoms even months after mild SARS-CoV-2 infections, although less frequently than adults (Buonsenso, 2022).

To standardize symptom tracking and develop unified approaches to the assessment of long COVID-19, the International Severe Acute Respiratory and Emerging Infection Consortium (ISARIC) developed a questionnaire for global research on the long-term outcomes of COVID-19 in children. Studies in various countries using this tool have shown that long COVID symptoms were observed in 24–35% of children (Buonsenso, 2022; Osmanov, 2022).

However, to date, the risk factors for the development of long COVID-19 in children remain poorly understood. As far as we know, no studies have been conducted in Ukraine to determine the prevalence, course characteristics, quality of life, and risk factors for long COVID-19 in children — an issue that becomes even more significant under the current conditions of martial law. The roles of vitamin D and zinc in the development of long COVID-19 have also not been established. These facts highlight the need for further research into long COVID-19 in children.

**3. Aim, main objectives, and their relevance**

**1) Scientific hypothesis**

Identification of clinical and psychological symptoms of long COVID in children during wartime will allow for timely detection of long COVID; assessing children's quality of life will help determine its impact on the medical and social aspects of their lives; identifying risk factors will enable timely preventive and therapeutic-rehabilitation interventions to prevent the development of long COVID symptoms and to reduce their impact on children's quality of life during wartime, as well as to avoid the development of more severe diseases in the future. Additionally, data will be obtained on the prevalence of long COVID in Ukraine.

**2) Aim and objectives**

The aim of our study is to improve the quality of life of children with long COVID-19 during wartime by identifying the clinical and psychosocial consequences of acute SARS-CoV-2 infection, as well as studying the prevalence and risk factors of long COVID in children.

**Research objectives:**

- To adapt the questionnaire of the International Severe Acute Respiratory and Emerging Infection Consortium (ISARIC) for use in Ukraine;
- To study the clinical course of COVID-19 in children during wartime;
- To identify the presence of long COVID symptoms in children using the adapted ISARIC “Paediatric COVID-19 Health and Wellbeing Follow-up” questionnaire;
- To assess the quality of life of children with long COVID during wartime using the Ukrainian version of the validated Pediatric Quality of Life Inventory-4.0-Core-All (PedsQL™ 4.0) questionnaire;
- To assess the mental health of children with long COVID;
- To investigate the status of vitamin D and zinc in children with long COVID;
- To determine immune system status in patients with long COVID;
- To identify risk factors for long COVID;
- To develop therapeutic and rehabilitation programs for children with long COVID to reduce its impact on their quality of life.

**3) Expected novelty of the research**

This study will be the first to investigate the symptoms and prevalence of long COVID in children in Ukraine. Additionally, it will identify the specific features of long COVID during wartime and evaluate its impact on children’s quality of life. For the first time, the role of vitamin D and zinc in the development of long COVID will be assessed. Based on the study results, a diagnostic and monitoring algorithm for long COVID will be developed, along with therapeutic and preventive measures to mitigate serious health consequences in children.

**4. Study design, methods, and research features**

**1) Study design**

We plan to conduct an observational study (with parental consent) involving 300 children from the Ternopil region who were diagnosed with COVID-19 during wartime.

Using the adapted ISARIC “Paediatric COVID-19 Health and Wellbeing Follow-up” questionnaire, a prospective long-term observational study will be carried out to identify short-term and long-term consequences of COVID-19 in children who previously received outpatient or inpatient treatment.

Quality of life will be assessed using the Ukrainian version of the validated Pediatric Quality of Life Inventory-4.0-Core-All (PedsQL™ 4.0) questionnaire. Additionally, we will investigate vitamin D and zinc status, and evaluate immune system functioning in the context of long COVID.

The observation will be conducted in several stages following the onset of initial symptoms: at 1 month, 3 months, 6 months, and 12 months.

Comprehensive assessment of the health status of children with COVID-19, determination of vitamin D and zinc levels in blood serum

1 month later

Survey of children using the adapted ISARIC questionnaire "Monitoring the health and well-being of children with COVID-19"

No symptoms of long COVID

Patients with symptoms of long COVID

3 months later

Control group

Survey of children using the adapted ISARIC questionnaire

Present symptoms of long COVID

No symptoms of long COVID

Comprehensive examination of children and assessment of quality of life

Assessment of children’s quality of life and determination of vitamin D and zinc levels in blood serum

6 months later

Comprehensive examination of children and assessment of quality of life, determination of vitamin D and zinc levels in blood serum. Immunological examination of children

1 year later

Comprehensive examination of children and assessment of quality of life

Figure. Study design

**2) Object of the study:**

The clinical course, quality of life, and psychological state of children with long COVID.

**3) Research methods:**

- **Clinical methods:** medical history collection and physical examination;
- **Questionnaires:** using the adapted ISARIC “Paediatric COVID-19 Health and Wellbeing Follow-up” questionnaire and the Ukrainian version of the validated Pediatric Quality of Life Inventory-4.0-Core-All (PedsQL™ 4.0) questionnaire;
- **General laboratory tests:** complete blood count including leukocytes, lymphocytes, neutrophils, erythrocytes, hemoglobin, platelets, erythrocyte sedimentation rate (ESR);
- **Biochemical tests:** measurement of D-dimer, C-reactive protein (CRP), alanine aminotransferase (ALT), aspartate aminotransferase (AST), total protein, procalcitonin, vitamin D, and zinc levels;
- **Immunological tests:** analysis of CD3+, CD4+, CD8+, and CD19+ lymphocyte subpopulations, and levels of immunoglobulins A, M, G, and E;
- **Mathematical and statistical analysis.**

**4) Study endpoints (effectiveness indicators):**

- Identification of long COVID symptoms in children using the adapted ISARIC “Paediatric COVID-19 Health and Wellbeing Follow-up” questionnaire;
- Determination of risk factors for long COVID;
- Early detection of psychological status disorders;
- Referral for specialized medical care;
- Implementation of preventive measures to avoid serious long-term complications;
- Development of therapeutic and rehabilitation programs for children with long COVID to reduce its impact on their quality of life.

**5. Expected results and practical value for the economy, society, and national security**

Timely detection of long COVID and the provision of effective care—taking into account children’s vitamin and mineral status as well as their psychological characteristics (in accordance with the developed rehabilitation program)—will help shorten the duration of illness and reduce the economic burden on the healthcare system. This is especially important under the limited-resource conditions of wartime. Furthermore, it will improve the quality of life of affected children, contributing to the development of a healthier younger generation in the post-war period.

As a result of this study, the public healthcare system will receive a simple, accessible, and effective method for the early diagnosis of psychological disorders in children with long COVID-19.

Such pathological conditions in children will be identified in a timely manner, enabling preventive measures to protect them from developing severe mental illnesses in the future and ensuring they receive appropriate qualified treatment.
