# Supplementary material for: Prevalence and duration of clinical symptoms of pediatric long COVID: findings from a one-year prospective study
Source: Front Pediatr. 2025 Sep 22;13:1645228. doi: 10.3389/fped.2025.1645228 (PMC12499359; doi:10.3389/fped.2025.1645228)
Supplement: Supplementary file 6 [file Datasheet3.pdf]

Витяг з протоколу № 70
засідання комісії з біоетики Тернопільського національного медичного університету імені І.Я. Горбачевського МОЗ України
від 01 серпня 2022 року

Присутні: проф. С.Н. Вадзюк (голова), проф. В.Б. Гощинський, проф. Я.Я. Боднар, проф. Ю.І. Бондаренко, проф. І.Я. Дзюбановський, директор обласного еколого-натуралістичного центру І.І. Герц, Галещук Ю.В. (секретар комісії).

Розглянули: біотичну експертизу заявки на фінансування наукових досліджень і розробок на тему «Оцінка якості життя та психологічного стану дітей з тривалим COVID-19 в умовах воєнного часу». Керівник проекту: доктор медичних наук, професор Боярчук О.Р.

Постановили: біотичну експертизу заявки на фінансування наукових досліджень і розробок на тему «Оцінка якості життя та психологічного стану дітей з тривалим COVID-19 в умовах воєнного часу». Керівник проекту: доктор медичних наук, професор Боярчук О.Р., щодо обстеження пацієнтів, проведення лабораторних, наукових досліджень відповідає вимогам норм та принципам біоетики. При виконанні роботи передбачене дотримання правил безпеки пацієнтів, збережені права та канони людської гідності, а також, морально-етичні норми у відповідності до основних положень GSP (1996 р.) Конвенції Ради Європи про права людини та біомедицину (від 04.04.1997 р.) Гельсінської декларації Всесвітньої медичної асоціації про етичні принципи проведення наукових медичних досліджень за участю людини (1964-2000 рр.), і наказу МОЗ України № 281 від 01.11.2000 р., Declaration of Helsinki «World Medical Association Declaration of Helsinki Ethical Principles for Medical Research Involving Human Subjects» (2001 р.), етичного кодексу ученого України (2009 р.).

Голова комісії з біоетики
Тернопільського національного медичного
університету імені І. Я. Горбачевського
МОЗ України, доктор медичних наук,
професор, заслужений діяч науки
і техніки України С.Н. Вадзюк

Секретар комісії з біоетики Ю.В. Галещук

Тернопільський національний медичний університет імені І.Я. Горбачевського Міністерства охорони здоров'я України
02010830

Особистий підпис ____________
завіряю
Заступник ректора з кадрових питань
Тернопільського національного
медичного університету
