# Supplementary material for: Prevalence and duration of clinical symptoms of pediatric long COVID: findings from a one-year prospective study
Source: Front Pediatr. 2025 Sep 22;13:1645228. doi: 10.3389/fped.2025.1645228 (PMC12499359; doi:10.3389/fped.2025.1645228)
Supplement: Supplementary file 7 [file Datasheet4.docx]

**Extract from Protocol No. 70**
**Meeting of the Bioethics Committee of I.Ya. Horbachevsky Ternopil National Medical University**
**Ministry of Health of Ukraine**
**Dated August 1, 2022**

**Attendees:** Prof. S.N. Vadziuk (Chair), Prof. V.B. Hoshchynskyi, Prof. Ya.Ya. Bodnar, Prof. Yu.I. Bondarenko, Prof. I.Ya. Dziubanovskyi, Director of the Regional Environmental and Naturalistic Center I.I. Hertz, Y.V. Haleshchuk (Secretary of the Committee).

**Reviewed:** Bioethical examination of the application for funding scientific research and development on the topic: **"Assessment of quality of life and psychological state of children with long-term COVID-19 under wartime conditions."** Project Leader: Doctor of Medical Sciences, Professor O.R. Boyarchuk.

**Resolution:** The bioethical examination of the application for funding scientific research and development on the topic: **"Assessment of quality of life and psychological state of children with long-term COVID-19 under wartime conditions,"** including patient examinations, laboratory and scientific studies, complies with the standards and principles of bioethics. The study ensures adherence to patient safety regulations, the protection of human rights and dignity, and compliance with moral and ethical standards in accordance with the key provisions of: Good Scientific Practice (GSP, 1996), The Council of Europe Convention on Human Rights and Biomedicine (04.04.1997), The Declaration of Helsinki of the World Medical Association on ethical principles for medical research involving human subjects (1964–2000), Order No. 281 of the Ministry of Health of Ukraine dated 01.11.2000, The Declaration of Helsinki “World Medical Association Declaration of Helsinki Ethical Principles for Medical Research Involving Human Subjects” (2001), The Ethical Code of the Scientist of Ukraine (2009).
